# 2,2′-(Disulfanediyl)dibenzoic acid–*N*,*N*′-bis­(3-pyridyl­meth­yl)ethane­diamide (1/1)

**DOI:** 10.1107/S1600536810036494

**Published:** 2010-09-18

**Authors:** Hadi D. Arman, Tyler Miller, Pavel Poplaukhin, Edward R. T. Tiekink

**Affiliations:** a Department of Chemistry, The University of Texas at San Antonio, One UTSA Circle, San Antonio, Texas 78249-0698, USA; b Chemical Abstracts Service, 2540 Olentangy River Rd, Columbus, Ohio 43202, USA; c Department of Chemistry, University of Malaya, 50603 Kuala Lumpur, Malaysia

## Abstract

The asymmetric unit of the title cocrystal, C_14_H_14_N_4_O_2_·C_14_H_10_O_4_S_2_, comprises a twisted 2,2′-(disulfanediyl)dibenzoic acid mol­ecule [dihedral angle between the benzene rings = 76.35 (10)°] and a U-shaped *N*,*N*′-bis­(3-pyridyl­meth­yl)ethane­diamide mol­ecule with the pyridyl groups lying to the same side of the central diamide moiety [C—C—C—N = 113.8 (2) and −117.6 (2)°]. The latter aggregate into supra­molecular tapes propagating along the *a* axis *via* centrosymmetric eight-membered amide {⋯OCNH}_2_ synthons. Intra­molecular N—H⋯O hydrogen bonds are observed. The 2,2′-(disulfanediyl)dibenzoic acid mol­ecules form carbox­yl–pyridine O—H⋯N hydrogen bonds, bridging a pyridine residue below the plane of the tape and one above the plane with two inter­vening *N*,*N*′-bis­(3-pyridyl­meth­yl)ethane­diamide mol­ecules. The supra­molecular chains are consolidated in the crystal packing by C—H⋯O contacts. An inter­molecular C—H⋯S inter­action also occurs.

## Related literature

For related studies on co-crystal formation involving 2-[(2-carb­oxy­phen­yl)disulfan­yl]benzoic acid, see: Broker & Tiekink (2007 ▶, 2010 ▶); Broker *et al.* (2008 ▶); Arman *et al.* (2010 ▶). For crystal engineering studies on *N*,*N*′-bis­(3-pyridyl­meth­yl)ethane­diamide, see: Poplaukhin & Tiekink (2010 ▶).
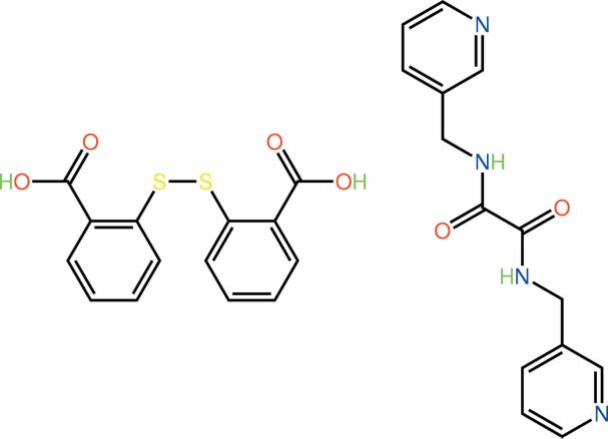


## Experimental

### 

#### Crystal data

C_14_H_14_N_4_O_2_·C_14_H_10_O_4_S_2_
*M* *_r_* = 576.63
Triclinic, 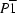

*a* = 10.015 (3) Å
*b* = 10.310 (3) Å
*c* = 14.795 (4) Å
α = 86.910 (16)°
β = 78.052 (15)°
γ = 69.554 (10)°
*V* = 1400.1 (7) Å^3^
*Z* = 2
Mo *K*α radiation
μ = 0.24 mm^−1^
*T* = 98 K
0.50 × 0.19 × 0.10 mm

#### Data collection

Rigaku AFC12/SATURN724 diffractometer
Absorption correction: multi-scan (*ABSCOR*; Higashi, 1995 ▶) *T*_min_ = 0.755, *T*_max_ = 1.000
10806 measured reflections
6365 independent reflections
5644 reflections with *I* > 2σ(*I*)
*R*_int_ = 0.031

#### Refinement

*R*[*F* ^2^ > 2σ(*F* ^2^)] = 0.051
*wR*(*F* ^2^) = 0.120
*S* = 1.09
6365 reflections
373 parameters
4 restraints
H atoms treated by a mixture of independent and constrained refinement
Δρ_max_ = 0.36 e Å^−3^
Δρ_min_ = −0.41 e Å^−3^

### 

Data collection: *CrystalClear* (Molecular Structure Corporation & Rigaku, 2005 ▶); cell refinement: *CrystalClear*; data reduction: *CrystalClear*; program(s) used to solve structure: *SHELXS97* (Sheldrick, 2008 ▶); program(s) used to refine structure: *SHELXL97* (Sheldrick, 2008 ▶); molecular graphics: *ORTEPII* (Johnson, 1976 ▶) and *DIAMOND* (Brandenburg, 2006 ▶); software used to prepare material for publication: *publCIF* (Westrip, 2010 ▶).

## Supplementary Material

Crystal structure: contains datablocks global, I. DOI: 10.1107/S1600536810036494/hg2712sup1.cif

Structure factors: contains datablocks I. DOI: 10.1107/S1600536810036494/hg2712Isup2.hkl

Additional supplementary materials:  crystallographic information; 3D view; checkCIF report

## Figures and Tables

**Table 1:** Hydrogen-bond geometry (Å, °)

*D*—H⋯*A*	*D*—H	H⋯*A*	*D*⋯*A*	*D*—H⋯*A*
N2—H1*n*⋯O2	0.88 (1)	2.37 (2)	2.736 (2)	105 (1)
N2—H1*n*⋯O2^i^	0.88 (1)	2.03 (1)	2.789 (3)	144 (2)
N3—H2*n*⋯O1	0.88 (1)	2.37 (2)	2.698 (2)	103 (1)
N3—H2*n*⋯O1^ii^	0.88 (1)	1.97 (1)	2.773 (3)	151 (2)
O4—H1*o*⋯N1^iii^	0.84 (2)	1.83 (2)	2.664 (3)	175 (1)
O6—H2*o*⋯N4^ii^	0.84 (2)	1.80 (2)	2.641 (3)	179 (4)
C2—H2⋯O3^iv^	0.95	2.53	3.220 (3)	129
C3—H3⋯O1^v^	0.95	2.48	3.261 (3)	139
C9—H9b⋯S1^i^	0.99	2.73	3.370 (2)	123

Symmetry codes: (i) 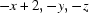; (ii) 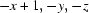; (iii) 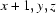; (iv) 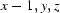; (v) 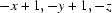.

**Fig. 1.**
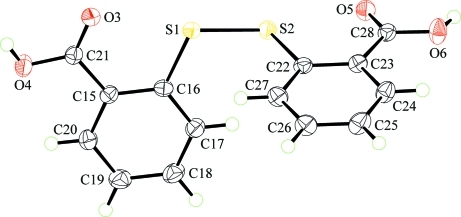
Molecular structure of 2-[(2-carboxyphenyl)disulfanyl]benzoic acid found in the structure of (I) showing atom-labelling scheme and displacement ellipsoids at the 50% probability level.

**Fig. 2.**
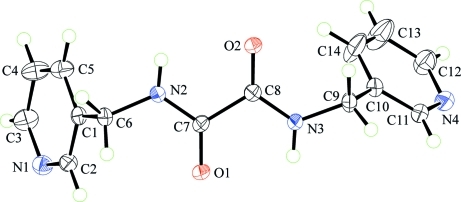
Molecular structure of N,N'-bis(3-pyridylmethyl)ethanediamide found in the structure of (I) showing atom-labelling scheme and displacement ellipsoids at the 50% probability level.

**Fig. 3.**
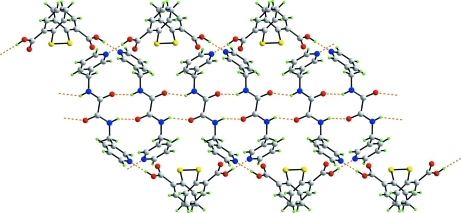
Supramolecular chain along the a axis in (I). The O—H···N and N—H···O hydrogen bonds are shown as orange dashed lines.

**Fig. 4.**
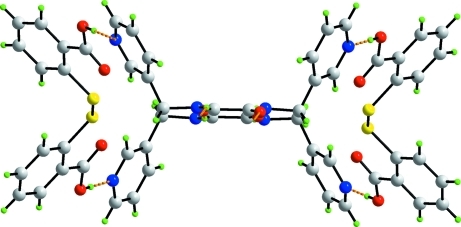
End-on view of the supramolecular chain along the a axis in (I). The O—H···N and N—H···O hydrogen bonds are shown as orange dashed lines.

**Fig. 5.**
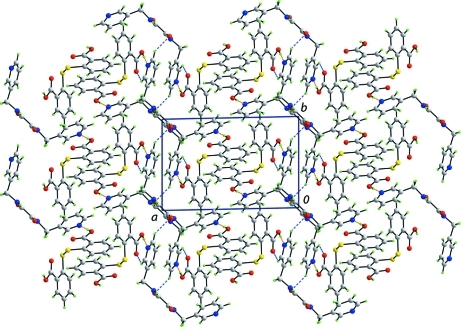
View in projection down the a axis in (I) showing the crystal packing. The O—H···N and N—H···O hydrogen bonds are shown as orange dashed lines, and C—H···O interactions are shown as blue dashed lines.

## supplementary crystallographic information

### Comment

As a continuation of studies into the phenomenon of co-crystallization of 2-[(2-carboxyphenyl)disulfanyl]benzoic acid (Broker & Tiekink, 2007; Broker *et al.*, 2008; Broker & Tiekink, 2010; Arman *et al.*, 2010), the co-crystallization of 2,2'-(disulfanediyl)dibenzoic acid and *N*,*N*'-bis(3-pyridylmethyl)ethanediamide (Poplaukhin & Tiekink, 2010) was investigated. The asymmetric unit of the resulting co-crystal contains one molecule of 2,2'-(disulfanediyl)dibenzoic acid, Fig. 1, and *N*,*N*'-bis(3-pyridylmethyl)ethanediamide, Fig. 2.

The 2,2'-(disulfanediyl)dibenzoic acid molecule adopts the expected conformation (Broker & Tiekink, 2007), stabilized in part by two close *S*···*O*(carbonyl) interactions, *i.e*. S1···O3 = 2.6520 (18) Å and S2···O5 = 2.6593 (19) Å; the dihedral angle formed between the benzene rings = 76.35 (10) °. The *N*,*N*'-bis(3-pyridylmethyl)ethanediamide molecule adopts a U-shape with the pyridyl groups lying to the same side of the central diamide moiety [C2—C1—C6—N2 = 113.8 (2) ° and N3—C9—C10—C11 = -117.6 (2) °]; the dihedral angle formed between the pyridyl rings = 72.24 (12) °. The pyridine-N atoms are each directed to the same side of the molecule.

Supramolecular tapes are formed comprising alternately orientated U-shaped *N*,*N*'-bis(3-pyridylmethyl)ethanediamide molecules and mediated by centrosymmetric eight-membered amide {···OCNH}_2_ synthons, Fig. 3; intramolecular N—H···O contacts are also noted, Table 1. This arrangement results in successive pairs of pyridine residues of the *N*,*N*'-bis(3-pyridylmethyl)oxamide molecules being orientated above and below the plane of the tape. The 2,2'-(disulfanediyl)dibenzoic acid molecules form carboxylic acid-OH···*N*-pyridine interactions so that a bridge is formed between a pyridine residue below the plane of the tape and one above the plane with two intervening *N*,*N*'-bis(3-pyridylmethyl)oxamide molecules. In summary, amide-mediated chains are gird by *N*,*N*'-bis(3-pyridylmethyl)oxamide molecules as highlighted in the end-on view shown in Fig. 4. The tapes are orientated along the *a* direction with the most prominent connection between them being of the type C—H···O, Fig. 5.

### Experimental

Equimolar amounts of 2-[(2-carboxyphenyl)disulfanyl]benzoic acid (Fluka) and *N*,*N*'-bis(3-pyridylmethyl)ethanediamide (Poplaukhin & Tiekink, 2010) were dissolved in a 1:1 ethanol/chloroform mixture. Crystals were harvested after a few days of slow evaporation.

### Refinement

C-bound H-atoms were placed in calculated positions (C–H 0.95–0.99 Å) and were included in the refinement in the riding model approximation with *U*_iso_(H) set to 1.2–1.5*U*_eq_(C). The O– and N-bound H-atoms were located in a difference Fourier map and were refined with distance restraints of O–H 0.840±0.001 Å and N—H = 0.880±0.001 Å, and with *U*_iso_(H) = *yU*_eq_(carrier atom); *y* = 1.5 for O and *y* = 1.2 for N.

### Crystal data

**Table:**

C_14_H_14_N_4_O_2_·C_14_H_10_O_4_S_2_	*Z* = 2
*M**_r_* = 576.63	*F*(000) = 600
Triclinic, *P*1	*D*_x_ = 1.368 Mg m^−^^3^
Hall symbol: -P 1	Mo *K*α radiation, λ = 0.71069 Å
*a* = 10.015 (3) Å	Cell parameters from 5721 reflections
*b* = 10.310 (3) Å	θ = 2.2–40.6°
*c* = 14.795 (4) Å	µ = 0.24 mm^−^^1^
α = 86.910 (16)°	*T* = 98 K
β = 78.052 (15)°	Block, colourless
γ = 69.554 (10)°	0.50 × 0.19 × 0.10 mm
*V* = 1400.1 (7) Å^3^	

### Data collection

**Table:**

Rigaku AFC12K/SATURN724 diffractometer	6365 independent reflections
Radiation source: fine-focus sealed tube	5644 reflections with *I* > 2σ(*I*)
graphite	*R*_int_ = 0.031
ω scans	θ_max_ = 27.5°, θ_min_ = 2.2°
Absorption correction: multi-scan (*ABSCOR*; Higashi, 1995)	*h* = −10→13
*T*_min_ = 0.755, *T*_max_ = 1.000	*k* = −12→13
10806 measured reflections	*l* = −18→19

### Refinement

**Table:**

Refinement on *F*^2^	Primary atom site location: structure-invariant direct methods
Least-squares matrix: full	Secondary atom site location: difference Fourier map
*R*[*F*^2^ > 2σ(*F*^2^)] = 0.051	Hydrogen site location: inferred from neighbouring sites
*wR*(*F*^2^) = 0.120	H atoms treated by a mixture of independent and constrained refinement
*S* = 1.09	*w* = 1/[σ^2^(*F*_o_^2^) + (0.0472*P*)^2^ + 0.7689*P*] where *P* = (*F*_o_^2^ + 2*F*_c_^2^)/3
6365 reflections	(Δ/σ)_max_ = 0.001
373 parameters	Δρ_max_ = 0.36 e Å^−^^3^
4 restraints	Δρ_min_ = −0.41 e Å^−^^3^

### Special details

**Table:**

Geometry. All e.s.d.'s (except the e.s.d. in the dihedral angle between two l.s. planes) are estimated using the full covariance matrix. The cell e.s.d.'s are taken into account individually in the estimation of e.s.d.'s in distances, angles and torsion angles; correlations between e.s.d.'s in cell parameters are only used when they are defined by crystal symmetry. An approximate (isotropic) treatment of cell e.s.d.'s is used for estimating e.s.d.'s involving l.s. planes.
Refinement. Refinement of *F*^2^ against ALL reflections. The weighted *R*-factor *wR* and goodness of fit *S* are based on *F*^2^, conventional *R*-factors *R* are based on *F*, with *F* set to zero for negative *F*^2^. The threshold expression of *F*^2^ > σ(*F*^2^) is used only for calculating *R*-factors(gt) *etc*. and is not relevant to the choice of reflections for refinement. *R*-factors based on *F*^2^ are statistically about twice as large as those based on *F*, and *R*- factors based on ALL data will be even larger.

### Fractional atomic coordinates and isotropic or equivalent isotropic displacement parameters (Å^2^)

**Table:**

	*x*	*y*	*z*	*U*_iso_*/*U*_eq_	
S1	0.95625 (5)	0.47011 (5)	0.27148 (3)	0.02334 (12)	
S2	0.77520 (5)	0.41447 (5)	0.31055 (3)	0.02470 (12)	
O1	0.57298 (14)	0.09350 (15)	0.05444 (10)	0.0277 (3)	
O2	0.92729 (14)	−0.08648 (15)	−0.06739 (10)	0.0274 (3)	
O3	1.18914 (15)	0.52923 (14)	0.18911 (10)	0.0261 (3)	
O4	1.20535 (15)	0.73668 (15)	0.14860 (11)	0.0292 (3)	
H1o	1.2938 (7)	0.688 (2)	0.1330 (18)	0.044*	
O5	0.57421 (16)	0.29499 (15)	0.34728 (10)	0.0274 (3)	
O6	0.49044 (17)	0.21673 (18)	0.48313 (10)	0.0324 (4)	
H2o	0.437 (2)	0.194 (3)	0.4549 (17)	0.049*	
N1	0.48914 (18)	0.59287 (18)	0.10362 (12)	0.0262 (4)	
N2	0.78541 (17)	0.12250 (17)	0.06156 (11)	0.0222 (3)	
H1n	0.8801 (4)	0.093 (2)	0.0407 (14)	0.027*	
N3	0.70914 (17)	−0.09173 (17)	−0.08631 (12)	0.0231 (3)	
H2n	0.6141 (4)	−0.064 (2)	−0.0674 (15)	0.028*	
N4	0.67717 (19)	−0.14651 (19)	−0.39347 (12)	0.0283 (4)	
C1	0.6801 (2)	0.3730 (2)	0.10738 (13)	0.0212 (4)	
C2	0.5347 (2)	0.4577 (2)	0.12348 (13)	0.0233 (4)	
H2	0.4635	0.4178	0.1500	0.028*	
C3	0.5902 (2)	0.6486 (2)	0.06698 (16)	0.0332 (5)	
H3	0.5598	0.7446	0.0540	0.040*	
C4	0.7377 (2)	0.5712 (3)	0.04726 (17)	0.0363 (5)	
H4	0.8066	0.6133	0.0200	0.044*	
C5	0.7829 (2)	0.4320 (2)	0.06776 (15)	0.0293 (4)	
H5	0.8834	0.3773	0.0548	0.035*	
C6	0.7231 (2)	0.2239 (2)	0.13762 (13)	0.0222 (4)	
H6A	0.7947	0.2082	0.1778	0.027*	
H6B	0.6360	0.2085	0.1750	0.027*	
C7	0.7054 (2)	0.06452 (19)	0.02830 (13)	0.0209 (4)	
C8	0.7929 (2)	−0.0458 (2)	−0.04751 (13)	0.0225 (4)	
C9	0.7676 (2)	−0.2038 (2)	−0.15588 (14)	0.0249 (4)	
H9A	0.7105	−0.2664	−0.1422	0.030*	
H9B	0.8694	−0.2581	−0.1517	0.030*	
C10	0.7639 (2)	−0.1524 (2)	−0.25316 (14)	0.0253 (4)	
C11	0.6821 (2)	−0.1873 (2)	−0.30620 (14)	0.0253 (4)	
H11	0.6266	−0.2430	−0.2794	0.030*	
C12	0.7536 (3)	−0.0680 (3)	−0.43111 (17)	0.0391 (5)	
H12	0.7508	−0.0393	−0.4930	0.047*	
C13	0.8371 (4)	−0.0266 (4)	−0.3833 (2)	0.0588 (8)	
H13	0.8907	0.0300	−0.4115	0.071*	
C14	0.8410 (3)	−0.0696 (3)	−0.29329 (19)	0.0517 (7)	
H14	0.8972	−0.0418	−0.2590	0.062*	
C15	0.9789 (2)	0.7291 (2)	0.23181 (13)	0.0219 (4)	
C16	0.8894 (2)	0.65541 (19)	0.27535 (13)	0.0220 (4)	
C17	0.7493 (2)	0.7285 (2)	0.32319 (14)	0.0269 (4)	
H17	0.6887	0.6794	0.3536	0.032*	
C18	0.6974 (2)	0.8720 (2)	0.32683 (16)	0.0321 (5)	
H18	0.6020	0.9204	0.3601	0.039*	
C19	0.7833 (2)	0.9455 (2)	0.28253 (16)	0.0327 (5)	
H19	0.7472	1.0438	0.2850	0.039*	
C20	0.9230 (2)	0.8740 (2)	0.23438 (14)	0.0273 (4)	
H20	0.9814	0.9243	0.2027	0.033*	
C21	1.1338 (2)	0.6551 (2)	0.18689 (13)	0.0221 (4)	
C22	0.7479 (2)	0.40288 (19)	0.43378 (13)	0.0220 (4)	
C23	0.6509 (2)	0.3389 (2)	0.48074 (13)	0.0223 (4)	
C24	0.6283 (2)	0.3319 (2)	0.57712 (14)	0.0279 (4)	
H24	0.5644	0.2871	0.6089	0.033*	
C25	0.6978 (2)	0.3893 (2)	0.62702 (15)	0.0315 (5)	
H25	0.6802	0.3855	0.6925	0.038*	
C26	0.7928 (2)	0.4519 (2)	0.58043 (15)	0.0293 (4)	
H26	0.8411	0.4909	0.6142	0.035*	
C27	0.8185 (2)	0.4583 (2)	0.48487 (15)	0.0274 (4)	
H27	0.8849	0.5009	0.4538	0.033*	
C28	0.5689 (2)	0.2819 (2)	0.43016 (14)	0.0230 (4)	

### Atomic displacement parameters (Å^2^)

**Table:**

	*U*^11^	*U*^22^	*U*^33^	*U*^12^	*U*^13^	*U*^23^
S1	0.0235 (2)	0.0226 (2)	0.0238 (3)	−0.00938 (18)	−0.00082 (18)	−0.00458 (17)
S2	0.0287 (3)	0.0311 (3)	0.0190 (2)	−0.0164 (2)	−0.00315 (19)	−0.00354 (18)
O1	0.0151 (6)	0.0334 (8)	0.0350 (8)	−0.0080 (6)	−0.0035 (6)	−0.0117 (6)
O2	0.0158 (6)	0.0346 (8)	0.0316 (8)	−0.0075 (6)	−0.0036 (6)	−0.0119 (6)
O3	0.0243 (7)	0.0246 (7)	0.0284 (8)	−0.0087 (6)	−0.0019 (6)	−0.0029 (6)
O4	0.0231 (7)	0.0279 (8)	0.0345 (8)	−0.0095 (6)	−0.0013 (6)	0.0058 (6)
O5	0.0319 (7)	0.0352 (8)	0.0198 (7)	−0.0171 (6)	−0.0054 (6)	−0.0021 (6)
O6	0.0339 (8)	0.0519 (10)	0.0230 (8)	−0.0280 (8)	−0.0084 (6)	0.0042 (7)
N1	0.0260 (8)	0.0275 (9)	0.0246 (9)	−0.0091 (7)	−0.0045 (7)	0.0016 (7)
N2	0.0174 (7)	0.0259 (8)	0.0224 (8)	−0.0060 (6)	−0.0026 (6)	−0.0078 (6)
N3	0.0165 (7)	0.0258 (8)	0.0271 (9)	−0.0065 (6)	−0.0036 (6)	−0.0092 (7)
N4	0.0274 (9)	0.0337 (9)	0.0264 (9)	−0.0136 (7)	−0.0053 (7)	−0.0017 (7)
C1	0.0234 (9)	0.0260 (9)	0.0156 (9)	−0.0097 (8)	−0.0041 (7)	−0.0029 (7)
C2	0.0229 (9)	0.0274 (10)	0.0206 (10)	−0.0106 (8)	−0.0030 (7)	−0.0009 (7)
C3	0.0323 (11)	0.0325 (11)	0.0360 (12)	−0.0133 (9)	−0.0082 (9)	0.0091 (9)
C4	0.0283 (11)	0.0410 (13)	0.0415 (13)	−0.0176 (10)	−0.0044 (9)	0.0127 (10)
C5	0.0221 (9)	0.0364 (11)	0.0295 (11)	−0.0105 (8)	−0.0059 (8)	0.0048 (9)
C6	0.0227 (9)	0.0259 (9)	0.0186 (9)	−0.0086 (7)	−0.0038 (7)	−0.0046 (7)
C7	0.0183 (8)	0.0229 (9)	0.0232 (10)	−0.0078 (7)	−0.0063 (7)	−0.0020 (7)
C8	0.0198 (9)	0.0254 (9)	0.0229 (10)	−0.0074 (7)	−0.0047 (7)	−0.0035 (7)
C9	0.0245 (9)	0.0247 (9)	0.0262 (10)	−0.0069 (8)	−0.0069 (8)	−0.0085 (8)
C10	0.0244 (9)	0.0281 (10)	0.0241 (10)	−0.0097 (8)	−0.0037 (8)	−0.0069 (8)
C11	0.0249 (9)	0.0293 (10)	0.0242 (10)	−0.0125 (8)	−0.0035 (8)	−0.0043 (8)
C12	0.0478 (14)	0.0460 (14)	0.0329 (13)	−0.0273 (12)	−0.0095 (10)	0.0035 (10)
C13	0.082 (2)	0.080 (2)	0.0464 (16)	−0.0664 (19)	−0.0207 (15)	0.0162 (15)
C14	0.0701 (19)	0.076 (2)	0.0388 (14)	−0.0562 (17)	−0.0209 (13)	0.0040 (13)
C15	0.0218 (9)	0.0252 (9)	0.0187 (9)	−0.0076 (7)	−0.0046 (7)	−0.0005 (7)
C16	0.0248 (9)	0.0230 (9)	0.0190 (9)	−0.0079 (7)	−0.0057 (7)	−0.0022 (7)
C17	0.0247 (10)	0.0277 (10)	0.0264 (11)	−0.0080 (8)	−0.0027 (8)	−0.0001 (8)
C18	0.0261 (10)	0.0280 (11)	0.0333 (12)	−0.0020 (8)	0.0004 (8)	−0.0006 (8)
C19	0.0372 (12)	0.0220 (10)	0.0323 (12)	−0.0042 (9)	−0.0034 (9)	0.0013 (8)
C20	0.0298 (10)	0.0251 (10)	0.0262 (10)	−0.0107 (8)	−0.0030 (8)	0.0037 (8)
C21	0.0232 (9)	0.0276 (10)	0.0175 (9)	−0.0108 (8)	−0.0044 (7)	−0.0003 (7)
C22	0.0228 (9)	0.0231 (9)	0.0197 (9)	−0.0073 (7)	−0.0036 (7)	−0.0038 (7)
C23	0.0207 (9)	0.0256 (9)	0.0212 (10)	−0.0073 (7)	−0.0062 (7)	−0.0015 (7)
C24	0.0250 (10)	0.0384 (11)	0.0219 (10)	−0.0129 (9)	−0.0046 (8)	0.0003 (8)
C25	0.0325 (11)	0.0457 (13)	0.0180 (10)	−0.0143 (10)	−0.0069 (8)	−0.0015 (9)
C26	0.0301 (10)	0.0372 (11)	0.0253 (11)	−0.0139 (9)	−0.0108 (8)	−0.0039 (8)
C27	0.0272 (10)	0.0312 (10)	0.0277 (11)	−0.0126 (8)	−0.0086 (8)	−0.0030 (8)
C28	0.0195 (9)	0.0252 (9)	0.0240 (10)	−0.0076 (7)	−0.0031 (7)	−0.0030 (7)

### Geometric parameters (Å, °)

**Table:**

S1—C16	1.790 (2)	C9—C10	1.510 (3)
S1—S2	2.0514 (9)	C9—H9A	0.9900
S2—C22	1.791 (2)	C9—H9B	0.9900
O1—C7	1.233 (2)	C10—C14	1.376 (3)
O2—C8	1.237 (2)	C10—C11	1.386 (3)
O3—C21	1.222 (2)	C11—H11	0.9500
O4—C21	1.321 (2)	C12—C13	1.382 (3)
O4—H1o	0.841 (15)	C12—H12	0.9500
O5—C28	1.219 (2)	C13—C14	1.384 (4)
O6—C28	1.319 (2)	C13—H13	0.9500
O6—H2o	0.84 (2)	C14—H14	0.9500
N1—C3	1.339 (3)	C15—C20	1.400 (3)
N1—C2	1.343 (3)	C15—C16	1.407 (3)
N2—C7	1.331 (2)	C15—C21	1.494 (3)
N2—C6	1.459 (2)	C16—C17	1.395 (3)
N2—H1n	0.880 (12)	C17—C18	1.387 (3)
N3—C8	1.328 (2)	C17—H17	0.9500
N3—C9	1.464 (2)	C18—C19	1.382 (3)
N3—H2n	0.880 (13)	C18—H18	0.9500
N4—C12	1.326 (3)	C19—C20	1.389 (3)
N4—C11	1.342 (3)	C19—H19	0.9500
C1—C5	1.386 (3)	C20—H20	0.9500
C1—C2	1.390 (3)	C22—C27	1.396 (3)
C1—C6	1.514 (3)	C22—C23	1.407 (3)
C2—H2	0.9500	C23—C24	1.399 (3)
C3—C4	1.388 (3)	C23—C28	1.489 (3)
C3—H3	0.9500	C24—C25	1.388 (3)
C4—C5	1.383 (3)	C24—H24	0.9500
C4—H4	0.9500	C25—C26	1.381 (3)
C5—H5	0.9500	C25—H25	0.9500
C6—H6A	0.9900	C26—C27	1.386 (3)
C6—H6B	0.9900	C26—H26	0.9500
C7—C8	1.534 (3)	C27—H27	0.9500
			
C16—S1—S2	105.30 (7)	N4—C12—C13	122.2 (2)
C22—S2—S1	105.35 (7)	N4—C12—H12	118.9
C21—O4—H1O	108 (2)	C13—C12—H12	118.9
C28—O6—H2O	113 (2)	C12—C13—C14	118.5 (2)
C3—N1—C2	117.81 (18)	C12—C13—H13	120.8
C7—N2—C6	121.95 (16)	C14—C13—H13	120.8
C7—N2—H1N	119 (2)	C10—C14—C13	120.4 (2)
C6—N2—H1N	119 (2)	C10—C14—H14	119.8
C8—N3—C9	123.05 (16)	C13—C14—H14	119.8
C8—N3—H2N	122 (2)	C20—C15—C16	119.16 (18)
C9—N3—H2N	114 (2)	C20—C15—C21	119.88 (17)
C12—N4—C11	118.65 (18)	C16—C15—C21	120.90 (17)
C5—C1—C2	117.70 (18)	C17—C16—C15	119.16 (18)
C5—C1—C6	121.89 (18)	C17—C16—S1	120.49 (15)
C2—C1—C6	120.31 (17)	C15—C16—S1	120.34 (15)
N1—C2—C1	123.64 (18)	C18—C17—C16	120.66 (19)
N1—C2—H2	118.2	C18—C17—H17	119.7
C1—C2—H2	118.2	C16—C17—H17	119.7
N1—C3—C4	122.4 (2)	C19—C18—C17	120.6 (2)
N1—C3—H3	118.8	C19—C18—H18	119.7
C4—C3—H3	118.8	C17—C18—H18	119.7
C5—C4—C3	119.1 (2)	C18—C19—C20	119.35 (19)
C5—C4—H4	120.4	C18—C19—H19	120.3
C3—C4—H4	120.4	C20—C19—H19	120.3
C4—C5—C1	119.31 (19)	C19—C20—C15	121.01 (19)
C4—C5—H5	120.3	C19—C20—H20	119.5
C1—C5—H5	120.3	C15—C20—H20	119.5
N2—C6—C1	114.21 (16)	O3—C21—O4	123.62 (18)
N2—C6—H6A	108.7	O3—C21—C15	121.66 (17)
C1—C6—H6A	108.7	O4—C21—C15	114.67 (17)
N2—C6—H6B	108.7	C27—C22—C23	118.96 (18)
C1—C6—H6B	108.7	C27—C22—S2	121.54 (16)
H6A—C6—H6B	107.6	C23—C22—S2	119.48 (14)
O1—C7—N2	124.84 (18)	C24—C23—C22	119.26 (17)
O1—C7—C8	121.27 (16)	C24—C23—C28	119.37 (18)
N2—C7—C8	113.89 (16)	C22—C23—C28	121.35 (17)
O2—C8—N3	125.32 (18)	C25—C24—C23	121.1 (2)
O2—C8—C7	121.87 (16)	C25—C24—H24	119.5
N3—C8—C7	112.80 (16)	C23—C24—H24	119.5
N3—C9—C10	113.04 (17)	C26—C25—C24	119.32 (19)
N3—C9—H9A	109.0	C26—C25—H25	120.3
C10—C9—H9A	109.0	C24—C25—H25	120.3
N3—C9—H9B	109.0	C25—C26—C27	120.63 (18)
C10—C9—H9B	109.0	C25—C26—H26	119.7
H9A—C9—H9B	107.8	C27—C26—H26	119.7
C14—C10—C11	117.0 (2)	C26—C27—C22	120.76 (19)
C14—C10—C9	122.02 (18)	C26—C27—H27	119.6
C11—C10—C9	121.01 (18)	C22—C27—H27	119.6
N4—C11—C10	123.35 (19)	O5—C28—O6	123.38 (17)
N4—C11—H11	118.3	O5—C28—C23	122.65 (18)
C10—C11—H11	118.3	O6—C28—C23	113.97 (17)
			
C16—S1—S2—C22	−88.85 (9)	C20—C15—C16—S1	178.78 (15)
C3—N1—C2—C1	−0.6 (3)	C21—C15—C16—S1	−4.0 (2)
C5—C1—C2—N1	−0.7 (3)	S2—S1—C16—C17	16.77 (17)
C6—C1—C2—N1	175.91 (17)	S2—S1—C16—C15	−164.61 (14)
C2—N1—C3—C4	1.6 (3)	C15—C16—C17—C18	1.0 (3)
N1—C3—C4—C5	−1.4 (4)	S1—C16—C17—C18	179.59 (17)
C3—C4—C5—C1	0.1 (3)	C16—C17—C18—C19	0.5 (3)
C2—C1—C5—C4	0.9 (3)	C17—C18—C19—C20	−0.3 (3)
C6—C1—C5—C4	−175.63 (19)	C18—C19—C20—C15	−1.4 (3)
C7—N2—C6—C1	−96.1 (2)	C16—C15—C20—C19	2.9 (3)
C5—C1—C6—N2	−69.8 (2)	C21—C15—C20—C19	−174.42 (19)
C2—C1—C6—N2	113.8 (2)	C20—C15—C21—O3	174.69 (18)
C6—N2—C7—O1	3.2 (3)	C16—C15—C21—O3	−2.5 (3)
C6—N2—C7—C8	−176.49 (17)	C20—C15—C21—O4	−2.8 (3)
C9—N3—C8—O2	3.2 (3)	C16—C15—C21—O4	179.98 (17)
C9—N3—C8—C7	−175.71 (17)	S1—S2—C22—C27	14.52 (18)
O1—C7—C8—O2	−172.03 (19)	S1—S2—C22—C23	−166.75 (14)
N2—C7—C8—O2	7.7 (3)	C27—C22—C23—C24	−0.3 (3)
O1—C7—C8—N3	6.9 (3)	S2—C22—C23—C24	−179.10 (15)
N2—C7—C8—N3	−173.41 (17)	C27—C22—C23—C28	178.05 (18)
C8—N3—C9—C10	−101.3 (2)	S2—C22—C23—C28	−0.7 (3)
N3—C9—C10—C14	62.5 (3)	C22—C23—C24—C25	1.3 (3)
N3—C9—C10—C11	−117.6 (2)	C28—C23—C24—C25	−177.13 (19)
C12—N4—C11—C10	−0.4 (3)	C23—C24—C25—C26	−1.3 (3)
C14—C10—C11—N4	1.1 (3)	C24—C25—C26—C27	0.3 (3)
C9—C10—C11—N4	−178.77 (18)	C25—C26—C27—C22	0.6 (3)
C11—N4—C12—C13	−0.3 (4)	C23—C22—C27—C26	−0.6 (3)
N4—C12—C13—C14	0.3 (5)	S2—C22—C27—C26	178.14 (16)
C11—C10—C14—C13	−1.1 (4)	C24—C23—C28—O5	173.73 (19)
C9—C10—C14—C13	178.8 (3)	C22—C23—C28—O5	−4.6 (3)
C12—C13—C14—C10	0.4 (5)	C24—C23—C28—O6	−6.2 (3)
C20—C15—C16—C17	−2.6 (3)	C22—C23—C28—O6	175.45 (18)
C21—C15—C16—C17	174.65 (18)		

### Hydrogen-bond geometry (Å, °)

**Table:**

*D*—H···*A*	*D*—H	H···*A*	*D*···*A*	*D*—H···*A*
N2—H1n···O2	0.88 (1)	2.37 (2)	2.736 (2)	105 (1)
N2—H1n···O2^i^	0.88 (1)	2.03 (1)	2.789 (3)	144 (2)
N3—H2n···O1	0.88 (1)	2.37 (2)	2.698 (2)	103 (1)
N3—H2n···O1^ii^	0.88 (1)	1.97 (1)	2.773 (3)	151 (2)
O4—H1o···N1^iii^	0.84 (2)	1.83 (2)	2.664 (3)	175 (1)
O6—H2o···N4^ii^	0.84 (2)	1.80 (2)	2.641 (3)	179 (4)
C2—H2···O3^iv^	0.95	2.53	3.220 (3)	129
C3—H3···O1^v^	0.95	2.48	3.261 (3)	139
C9—H9b···S1^i^	0.99	2.73	3.370 (2)	123

Symmetry codes: (i) −*x*+2, −*y*, −*z*; (ii) −*x*+1, −*y*, −*z*; (iii) *x*+1, *y*, *z*; (iv) *x*−1, *y*, *z*; (v) −*x*+1, −*y*+1, −*z*.
